# Root resorption pattern and root length of mandibular primary molars in children: a cross-sectional radiographic study

**DOI:** 10.1007/s40368-025-01052-3

**Published:** 2025-05-07

**Authors:** C. S. Garcete Delvalle, M. Bruna del Cojo, M. R. Mourelle Martínez, M. J. De Nova García

**Affiliations:** 1 Department of Dental Clinical Specialties, School of Dentistry, Faculty of Dentistry, Complutense University of Madrid, 28040 Madrid, Spain; 2 Department of Dentistry, School of Medicine, CEU San Pablo University, 28668 Madrid, Spain

**Keywords:** Root resorption pattern, Mandibular primary molar dentition, Primary molar resorption, Panoramic radiograph

## Abstract

**Purpose:**

To determine the root resorption pattern and calculate the root resorption length of mandibular primary molars in children.

**Methods:**

A cross-sectional and descriptive study was conducted of 367 panoramic radiographs of healthy nonorthodontic children, 169 girls with a mean age of 9.39 years and 198 boys with a mean age of 9.02 years. The length of the mesial and distal roots of the primary molar was calculated using a computer program (PixelStick®) that measures the pixels indicated in the image. Student’s t test, the Mann‒Whitney *U* test and the Kruskal‒Wallis test were used for comparisons, and *p* < 0.05 indicated statistical significance.

**Results:**

A 0.84 mm delay in root resorption of the first mandibular primary molar (74) in boys was described. There was also a delay in the resorption of the mesial root of 0.89 mm and 1.12 mm from the distal root of the second mandibular primary molar (75) in boys and a significant increase in root resorption in girls (*p* < 0.05). The mesial and distal roots of the first mandibular primary molar were resorbed equally; however, the mesial root of the second mandibular primary molar was resorbed before the distal root.

**Conclusions:**

Delays in root resorption were detected in boys, whereas advances in root resorption were detected in girls. The root resorption pattern of the first mandibular primary molar was symmetrical; however, the resorption pattern of the second mandibular primary molar was asymmetric, with the mesial root being resorbed first. Clinical and radiographic monitoring of mandibular primary molars with unevenly resorbed roots is recommended to avoid the complications associated with over retained molars.

**Supplementary Information:**

The online version contains supplementary material available at 10.1007/s40368-025-01052-3.

## Introduction

During the development of mixed dentition, complex events, such as the resorption of the roots of temporary teeth and the development of permanent dentition, occur. The chronology and coordination of these events allows the growth and development of the stomatognathic system (Marks et al. 1995).

The dental follicle surrounds the enamel organ of each tooth and originates in the mesenchyme of the neural crest. It regulates osteoclastogenesis and osteogenesis, which are necessary for the formation of the periodontal ligament and the development of the eruptive process. Two regions are described in the dental follicle: a coronal region and an apical or basal region. These regions are differentiated not only by location but also by the cellular events that occur (Wise 2009). In the coronal region, cells with osteoclastic capacity accumulate to resorb the alveolar bone and the roots of the temporary tooth; at the same time, the permanent tooth begins to move in an occlusal direction, and bone deposition occurs in the basal region (Zeng et al. 2022).

The roots of the temporary teeth are resorbed when the germ of the permanent tooth begins an active eruption, and the dental follicle approaches the root surface of the temporary teeth. If the location and direction differ, exfoliation of the temporary tooth does not occur (Harokopakis-Hajishengallis 2007). The premolar follicle develops below the divergent roots of the lower temporary molars. The position and size of the tooth germ affect the pattern of root resorption (Prove et al. 1992).

In 36% of the cases, one of the roots of the temporary molars is not resorbed, indicating an asymmetric root resorption pattern (Prove et al. 1992). The roots of the second temporary molars are very divergent and curved and are many times larger than the germ of the successor tooth. This could explain why there are cases in which one of the roots of a temporary molar has not been resorbed, resulting in dental retention. The mechanisms associated with the physiological root resorption of temporary teeth have been widely studied (Harokopakis-Hajishengallis 2007). However, few studies have analysed the root resorption pattern of mandibular temporary molars. The lack of resorption of one of the roots of temporary molars can lead to dental retention and ectopic eruptions, causing alterations in the chronology and development of the eruption.

Prolonged retention of the primary teeth may have many causes, such as impacted permanent teeth, an ectopic path of eruption, a congenitally absent premolar, ankylosis, or disruption of the normal exfoliation of the primary molars. A high incidence of ankylosis in the second mandibular molars has been documented. One factor that may predispose patients to tooth ankylosis of the primary molars is an asymmetric and disproportionate pattern of root reception (Eşian et al. 2022).

There are no studies that have analysed root resorption patterns and provided a table showing the average distal and mesial root lengths of mandibular primary molars by age and sex. This research could help paediatric dentists and orthodontics understand the exfoliation process of their patients’ primary teeth and, therefore, make clinical decisions. It could also be useful for future researchers.

The null hypothesis was that the distal and mesial roots of the primary mandibular molars reabsorbed symmetrically, and if there was an asymmetrical pattern of root resorption due to chance, the alternative hypothesis was that there was an asymmetrical root resorption pattern of the primary molars, and these differences were not due to chance. To test this hypothesis, the aim of this study was to determine the root resorption pattern and calculate the root resorption length of mandibular primary molars in children.

## Materials and methods

A cross-sectional and descriptive study was designed. It consisted of 367 panoramic radiographs of healthy nonorthodontic children. Two blinded operators evaluated the panoramic radiographs of the patients from the Department of Clinical Dental Specialties of the Complutense University of Madrid. The two operators were experienced dentists who were trained in dental radiography diagnosis, and 20 panoramic radiographs were measured per day. All guardians signed informed consent forms regarding the transfer of the data from their medical records, allowing their use for research purposes. The selected data were treated anonymously following the current data protection law. Before starting the research, this study was approved by the Ethics Committee of the San Carlos’ Clinical Hospital of Madrid.

The sample size was determined from the total number of paediatric patients who attended the Department of Clinical Dental Specialties at the Faculty of Dentistry of the Complutense University of Madrid between 2020 and 2024 using the Yamane’s formula, with a margin of error of 0.05 and a 95% confidence interval. The calculated sample size was 366.701 patients. Therefore, the sample consisted of 367 panoramic radiographs of healthy children between 6 and 11.9 years old without orthodontic treatment. The chronological age was calculated up to two decimal points for each child by subtracting the date of birth from the date of the X-ray after the age was converted to decimals.

Inclusion criteria:

- Healthy child patient,
- Spanish population,
- Age between 6 and 11.9 years,
- Informed consent of the guardian or legal representative.

Exclusion criteria:

- Absence of data regarding the date of X-ray collection,
- Distorted, irregular root apex and/or noncalibrated X-rays
- Caries, fillings, and pulp treatments of the lower temporary molars,
- Bilateral agenesis in quadrants III and IV,
- Patients who have received and/or are undergoing orthodontic treatment

After applying the inclusion and exclusion criteria mentioned above, the distribution of the 367 children according to age and sex is shown in Tables 1 and 2. The chronological age of the 367 healthy children was 9.19 (± 1.62); according to sex, the chronological age of the 169 females was 9.39 (± 1.45), and the mean age of the 198 males was 9.02 (± 1.74). These data indicate that the females are, on average, 0.37 years older than the males are.

**Table 1:** Distribution of the children

Sex	*N*	Asymmetry	Kurtosis	Norm. *p* value	Average	Median	Standard deviation	Minimum	Maximum	IQR
Girls	169	0.04	− 1.07	.000*	9.39	9.47	1.45	6.11	11.99	2.45
Boys	198	0.02	− 1.16	.000*	9.02	8.94	1.73	6.00	11.98	2.63

**Table 2:** Distribution of the children according to age and sex

Age group	Girls *n* (%)	Boys *n* (%)	Total *n* (%)
10–10.99	31 (8.44%)	31 (8.44%)	62 (16.89%)
11–11.99	30 (8.17%)	35 (9.53%)	65 (17.70%)
6–6.99	5 (1.3%)	37 (10.08%)	42 (11.38%)
7–7.99	35 (9.53%)	30 (8.17%)	65 (17.70%)
8–8.99	32 (8.71%)	33 (8.99%)	65 (17.70%)
9–9.99	36 (9.8%)	32 (8.71%)	68 (18.71%)
Total	169 (46%)	198 (53.95%)	367 (100%)

## Radiographic analysis

Most authors have studied root resorption in primary molars using the Haavikko et al. method (Haavikko 1973a, b), which studies the mesial and distal roots equally, not separately in a qualitative manner, establishing the stage of root resorption. Since the roots of biradicular teeth cannot be resorbed symmetrically in all cases (Peretz et al. 2013), in this study, the roots were studied separately via software (PixelStick®). The lengths of the mesial and distal roots of the primary molars were calculated using the selected software.

PixelStick is a tool for measuring distances, angles and colours on a screen. It is like an onscreen virtual ruler that can be used vertically, horizontally and at any angle to measure distances, angles and much more just by dragging. Using the palette, distances and angles can be locked (and also using the shift key). It supports scaling for Google Maps, Yahoo Maps, Photoshop and customised scaling options. It is a measuring tool that can be pinched and stretched to measure anything on a screen. The loupe is used to magnify anything on the screen.

To obtain the measurement in mm, it was necessary to obtain the relationship between the pixel size and mm. Once the cemento–enamel junction and the occlusal plane were located, we used the software as follows: the supplementary document (for details, see the supplementary document—calibration).

**Fig. 1:**
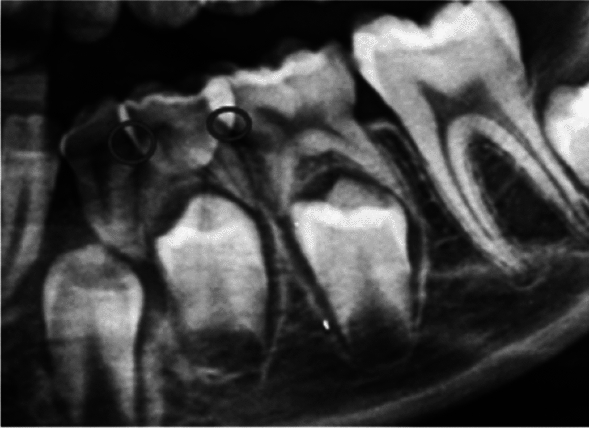
The location of the cemento-enamel junction of the first left primary molar is marked

First, the cemento-enamel junction was located (Fig. 1).

**Fig. 2:**
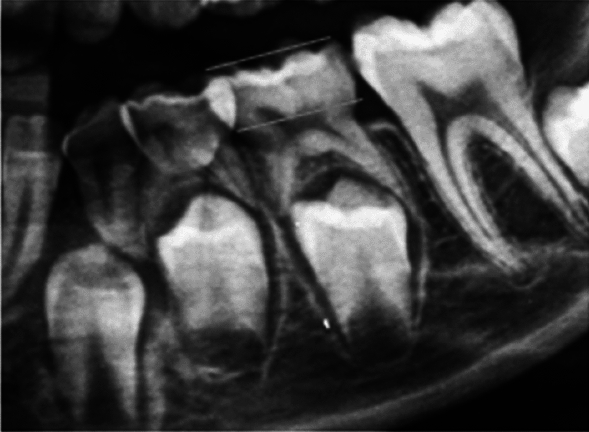
The anatomical crown forms the most occlusal part up to the cemento-enamel junction

The cemento-enamel line is the area where the crown’s convexity ends and where the radiopacity of the enamel is no longer visible. Following the identification of the cemento-enamel line, the occlusal plane was located, as shown in the following graph (Fig. 2).

**Fig. 3:**
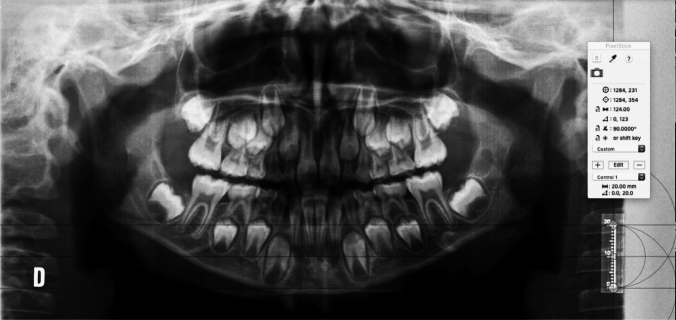
Checking the X-ray calibration. When the cursor is placed during the calibration, these parameters must match before moving on to the next step (20 mm)

**Fig. 4:**
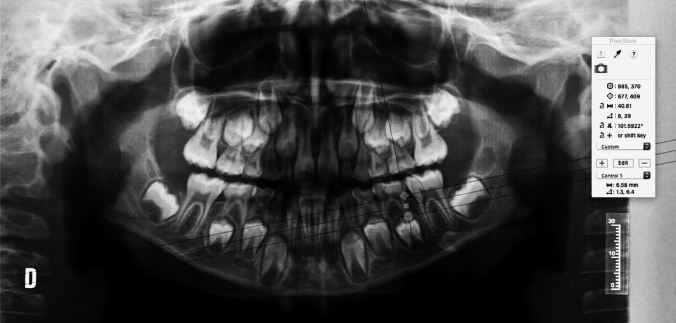
Estimated distal root length of the second mandibular primary molar. In the present case, it measures 6.58 mm (the distal root length was calculated as the distance between the distal cemento-enamel junction and the most apical point of the distal root)

**Fig. 5:**
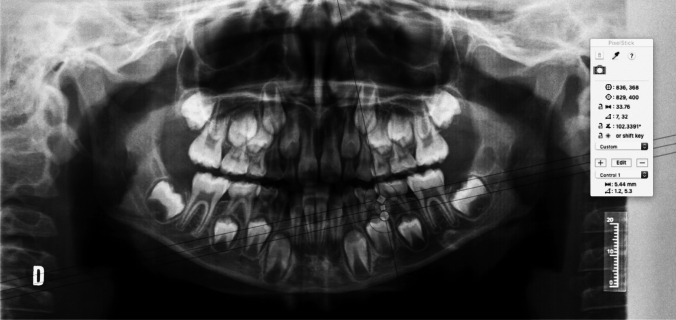
Estimated mesial root length of the second mandibular primary molar. In the present case, it measures 5.44 mm (the mesial root length was calculated as the distance between the distal cemento-enamel junction and the most apical point of the mesial root)

The following figures present an example of the methodology used to obtain the distal root length (DRL) of the second mandibular primary molar (75) and the mesial root length (MRL) of the second mandibular primary molar (75) (Figs. 3, 4, 5).

From the total sample of the study group (*n* = 367), 40 cases (10.9%) were randomly selected from among the 367 participants. Forty subjects were selected via the random sampling function implemented in SPSS statistics within their menus. This subsample (*N* = 40) was studied at two different times by the same evaluator and by 2 different evaluators. No differences in any of the average variables reached statistical significance (*p* > 0.05); that is, there were no differences between the measurements made by observer 1 with respect to those of observer 2. In addition, the correlation coefficients (which can be considered reliability coefficients) that were obtained were so high that they bordered on perfection (r = 1; maximum reliability). Therefore, there was very solid statistical evidence of a high degree of interobserver reliability, and the values recorded were independent of who made the measurements.

### Statistical analysis

The statistical analysis was carried out using the computer application IBM SPSS Statistics version 25 (reference: IBM Corp. Released 2017. IBM SPSS Statistics v 25.0 for Windows; Armonk. NY. USA).

The statistical techniques and tests applied consisted of the following:

- For the groups with *N* > 50, the Kolmogorov‒Smirnov test was applied, whereas for the groups with *N* < 50, the Shapiro‒Wilk test was used.
- The quantitative variables are described using the usual measures of (a) centrality (mean and median) and (b) variability (observed range, standard deviation and interquartile range).
- Student’s t test (parametric) was used when two independent samples were compared under the assumption of normality. For the comparison of the means among groups of different subjects (independent of each other), the nonparametric alternative of the Mann‒Whitney test was used since the variables did not meet normality criteria.
- The Wilcoxon test (nonparametric) was used when two sets of paired measurements (or repeated measures) were compared under the assumption of nonnormality. The Kruskal‒Wallis test (nonparametric) was used when more than two groups were compared while independence in the samples was considered under the assumption of nonnormality.
- The effect size was calculated to express the magnitude of the differences between groups. The effect size was expressed in R2 (scale: 0–1) so that it could be compared between different types of data (units of measurement) and between different statistical tests.

In all the inferential statistical tests, *p* < 0.05 was considered significant, and *p* < 0.01 was considered highly significant.

## Results

### Physiological root resorption of the mandibular molars

The centrality values of the mesial and distal lengths of the first temporary molar are similar to those shown in Table 3; however, the mesial and distal root lengths of the second temporary molar are different, indicating a possible asymmetric root resorption pattern.

**Table 3:** Centrality and dispersion values of the mesial and distal root lengths of temporary molars

Sex	*N*	Shape exploration			Centrality		Dispersion			
		Asymmetry	Kurtosis	Norm. *p* value	Average	Median	Standard deviation	Minimum	Maximum	IQR
MRL 74										
Girls	169	0.60	− 0.91	.000*	1.55	1.29	1.67	0.00	5.83	2.76
Boys	198	− 0.01	− 1.20	.000*	2.47	2.86	1.96	0.00	7.03	4.04
DRL 74										
Girls	169	0.48	− 1.12	.000*	1.63	1.53	1.70	0.00	6.29	3.08
Boys	198	− 0.17	− 1.42	.000*	2.54	3.06	1.98	0.00	6.49	4.15
MRL 75										
Girls	169	0.08	− 1.16	.000*	2.69	3.30	2.26	0.00	8.54	4.45
Boys	198	− 0.65	− 0.77	.000*	3.69	4.37	2.17	0.00	7.32	2.58
DRL 75										
Girls	169	− 0.08	− 1.52	.000*	3.28	4.00	2.71	0.00	8.00	5.53
Boys	198	− 0.95	− 0.49	.000*	4.52	5.42	2.47	0.00	7.81	2.74

NS: Non-significant deviation, the variable is normally distributed

*Significant, the variable is not normally distributed

When *n* > 50, the Kolmogorov–Smirnov normality test is used; when *n* < 50, the Shapiro–Wilk test is used

### Root length of the primary molars

#### First mandibular primary molar

The mean mesial root length (MRL) of the first mandibular primary molar (74) was 2.05 mm (± 1.89), and the mean distal root length (LDR) of the first mandibular primary molar (74) was 2.19 mm (± 1.92). The values of the root lengths are described in Table 4, stratified by age group and sex.

**Table 4:** Mean root length of the mesial and distal roots of the first mandibular primary molar (74) according to sex and age

74	Age	Girls (mm)	Boys (mm)
DRL 74	6–6.99	4.80 (± 0.92)	4.55 (± 0.90)
	7–7.99	3.37 (± 1.01)	3.67 (± 0.93)
	8–8.99	2.44 (± 1.35)	3.57 (± 1.03)
	9–9.99	1.15 (± 1.17)	1.95 (± 1.83)
	10–10.99	0.47 (± 1.17)	1.30 (± 1.67)
	11–11.99	0.00 (± 0.00)	0.09 (± 0.56)
MRL 74	6–6.99	5.02 (± 0.88)	4.58 (± 0.97)
	7–7.99	3.07 (± 1.12)	3.59 (± 1.12)
	8–8.99	2.41 (± 1.32)	3.46 (± 1.16)
	9–9.99	1.13 (± 1.31)	1.81 (± 1.57)
	10–10.99	0.38 (± 0.94)	1.22 (± 1.45)
	11–11.99	0.00 (± 0.00)	0.08 (± 0.49)

#### Second mandibular primary molar

The variability in the measurements, as shown by the standard deviations, underlines the individual differences in the root length of the second mandibular molar. The mean mesial root length (MRL) of the second mandibular primary molar (75) was 3.23 mm (± 2.27), and the mean distal root length (DRL) of the second mandibular primary molar (75) was 3.95 mm (± 2.66). The values of the root lengths are described in Table 5, stratified by age group and sex.

**Table 5:** The mean length of the mesial and distal roots of the second mandibular primary molar (75) according to sex and age

75	Age	Girls (mm)	Boys (mm)
DRL 75	6–6.99	7.75 (± 0.18)	6.26 (± 0.82)
	7–7.99	5.56 (± 1.35)	5.81 (± 1.14)
	8–8.99	4.59 (± 1.57)	5.74 (± 1.38)
	9–9.99	3.54 (± 2.53)	5.11 (± 1.66)
	10–10.99	1.50 (± 2.06)	3.47 (± 2.30)
	11–11.99	0.03 (± 0.18)	0.83 (± 1.82)
MRL 75	6–6.99	6.92 (± 1.76)	5.42 (± 0.96)
	7–7.99	4.53 (± 1.05)	4.79 (± 1.04)
	8–8.99	3.84 (± 1.24)	4.76 (± 1.29)
	9–9.99	2.60 (± 1.99)	3.71 (± 1.52)
	10–10.99	1.43 (± 1.93)	2.94 (± 2.19)
	11–11.99	0.03 (± 0.18)	0.57 (± 1.32)

### Root length according to sex

The mean root length of the mesial root of 74 was 1.55 mm (± 1.68), and that of the distal root was 1.63 mm (± 1.71) in girls. In boys, the mean length of the mesial root was 2.47 mm (± 1.96), and that of the distal root was 2.54 mm (± 1.99). This finding indicates a delay in root resorption of the mesial root of 0.92 mm (*p* < 0.05) and a delay in root resorption of the distal root of 0.91 mm (*p* < 0.05). Once the ages were equalised, a delay in root resorption of 0.84 mm was described for both the mesial and distal roots in boys, and these differences were statistically significant.

The values of the roots of 75 were greater. In girls, the average root length of the mesial root was 2.69 mm (± 2.26) and that of the distal root was 3.28 mm (± 2.72). In boys, the average values were 3.69 mm (± 2.18) for the mesial root and 4.52 mm (± 2.48) for the distal root. This finding indicates a delay in root resorption of the mesial root of 1 mm (*p* < 0.05) and a delay in root resorption of the distal root of 1.24 mm (*p* < 0.05). Once the ages were equalised, a delay in root resorption of 0.89 mm in the mesial root and 1.12 mm in the distal root was detected in boys, and these differences were statistically significant. Table 6 summarises the main differences according to sex.

**Table 6:** Comparative summary of the mesial and distal root lengths of the mandibular primary molars according to sex

	Girls (mm)	Boys (mm)	Difference (mm)	Difference once the ages were equalised (mm)	(*p*)	Delay or advance
MRL 74	1.55	2.47	0.92	0.84	< 0.05	Delay in root resorption (RR) of 0.84 mm in the mesial root of 74 in boys or advance in root resorption (RR) of 0.84 mm in the mesial root of 74 in girls
DRL 74	1.63	2.54	0.91	0.84	< 0.05	Delay in root resorption (RR) of 0.84 mm in the distal root of 74 in boys or advance in root resorption (RR) of 0.84 mm in the distal root of 74 in girls
MRL 75	2.69	3.69	1	0.9	< 0.05	Delay in RR of 0.89 mm in the distal root of 75 in boys or advance in root resorption (RR) of 0.89 mm in the distal root of 75 in girls
DRL 75	3.28	4.52	1.24	1.12	< 0.05	Delay in RR of 1.12 mm in the distal root of 75 in boys or advance in root resorption (RR) of 1.12 mm in the distal root of 75 in girls

A delay in root resorption was detected in boys, whereas advanced root resorption was detected in girls, and these differences were statistically significant (*p* < 0.05).

### Root resorption pattern of the mandibular primary molars

The central values for the differential measurements of the roots of the first mandibular primary molar were close to 0 in both mean and median. This finding indicates that there were no major differences in root resorption between the mesial and distal roots and that both roots were resorbed symmetrically. However, in the second mandibular primary molar, higher centrality measurements were described, indicating that the roots were resorbed differently, that is, asymmetrically. The values were negative, and the mesial root was resorbed before the distal root. According to the Mann‒Whitney *U* test, the difference between the mesial and distal root resorption of 74 indicates a symmetrical root resorption pattern, meaning that both types of roots were reabsorbed equally (*p* > 0.05). On the other hand, a statistically significant difference was observed between the resorption of the mesial and distal roots of 75, indicating that the mesial root was reabsorbed before the distal root, thus indicating an asymmetrical root pattern (*p* < 0.05).

## Discussion

In 1973, Haavikko et al. published the first study on the reliability of panoramic radiography in determining the stage of root resorption of primary dentition (Haavikko and Mattila 1973), and many authors have used this screening method for studying root resorption of primary dentition since then (Haavikko 1973a, b) (Caleya et al. 2022; Fulton and Liversidge 2016; Haralabakis et al. 1994). The third quadrant was selected for this study, since, according to various authors, it is the quadrant that suffers the least distortion and magnification (Kim et al. 2011; Philipp and Hurst 1978).

The role of the dental follicle in eruption was described and demonstrated by Cahill and Marks in 1980. In cases where the follicle was removed, tooth eruption did not occur, and in cases where it was maintained but a dental replica was introduced, the dental replica erupted (Marks and Cahill 1984). Over the years, it has been described in the literature that temporary molars without a permanent successor are also resorbed, but a delay in resorption has been described (Lin et al. 2012). In this study, to avoid bias related to the presence or absence of dental follicles, patients with agenesis of the lower premolars, caries, conservative treatments, and/or orthodontic treatments were excluded in order to study only the root resorption of temporary molars under equal conditions. In the scientific literature, an advance in the exfoliation of primary dentition is described in terms of the presence of caries, whether conservative or under orthodontic treatments (Ishikura 1991; Peretz et al. 2013). Many studies have shown that the effects of caries, pulp necrosis and pulpotomy can lead to premature exfoliation (Haralabakis et al. 1994).

The authors such as Haavikko et al. have extensively studied the root resorption of the primary dentition, but they have studied the mesial and distal roots together, so they have not been able to describe asymmetric patterns in the root resorption of the primary molars (Haavikko 1973a, b). The authors who studied the mesial and distal roots separately reported that, in 36% of the cases, asymmetric root resorption of the primary molars occurred (Prove et al. 1992). To avoid confusion in this study, we studied the mesial and distal roots of the primary molars separately using a software.

Peretz et al. reported that between 41 and 56% of root resorption of the mesial and distal roots of primary molars is symmetrical, so both types of roots are not resorbed with a symmetrical pattern in all cases (Peretz et al. 2013). In this study, we found that the mesial root of primary molars was resorbed 0.51 mm before the distal root; for 74, the difference was 0.05 mm. Regarding 75, this same finding is repeated, but the difference is greater (1.02 mm). This difference could be attributed to the discrepancy between the size of the successor premolar and the curvature of the roots of the second primary molar (Bhavyaa et al. 2022; Peretz et al. 2013). Another explanation could be the location of the successor germ, which is associated with the resorption of the root of the primary molar (Peretz et al. 2013).

We did not find any differences in the length of the mesial or distal roots of 74; thus, both types of roots were resorbed in a symmetrical pattern. However, the mesial and distal roots of the second mandibular primary molar exhibited an asymmetrical resorption pattern, and the mesial root was resorbed before the distal root. This clinical finding confirms the results found by other authors, who reported an asymmetrical pattern of root resorption in temporary molars (Peretz et al. 2013). Our results indicate that the mesial root is resorbed before the distal root, consistent with authors such as Moorrees et al. (1963). However, other authors reported that the distal root is resorbed before the distal root (Peretz et al. 2013). The difference may be attributed to different study methodologies. Moorrees et al. used lateral or oblique radiographs, Peretz et al. used periapical or bitewing radiographs, and we used panoramic radiographs. Moorrees et al. used 4 stages of root resorption (root resorbed 1/4; root resorbed 1/2; root resorbed 3/4 and exfoliation), whereas Peretz et al. used 3 stages of root resorption (< 1/4; 1/4–3/4, > 3/4), but both types of roots (mesial and distal) were studied together. We studied the roots separately and calculated the length of the root in mm; it was necessary to obtain the relationship between the pixel size and the scale (mm). This method was used previously by our team for studying the root resorption of primary molars in children with osteogenesis imperfecta medicated with bisphosphonates (Garcete Delvalle et al. 2025).

Systemic diseases that cause alterations in dental follicles, such as calcifying hyperplastic follicles, can lead to agenesis and even anodontia. This is because the dental follicles are atypical (Sandler et al. 1988). One of the clinical manifestations of Maroteaux–Lamy syndrome, known as mucopolysaccharidosis, is the lack of tooth eruption caused by abnormal follicles (Gomez et al. 1998). Moreover, antiresorptive therapy, such as bisphosphonates, can alter the eruptive process, resulting in a delay in the eruption and resorption of temporary teeth (Vuorimies et al. 2017). In this study, only children without systemic diseases were studied to only and exclusively determine the pattern of resorption of the mandibular primary molars.

An analysis of the root lengths of temporary molars according to sex revealed a delay of 0.84 mm in the root resorption of the first primary molar in boys. There was also a delay in the resorption of the mesial root of 0.89 mm and 1.12 mm from the distal root of the second primary molar in boys or advancement in girls, with statistical significance. This result is in accordance with the findings of previous authors regarding the differences between sexes, in which the eruptive process (dental development, root resorption of the primary dentition and dental eruption) was delayed in boys (Caleya et al. 2022; Garcete Delvalle et al. 2024; Haavikko 1973a, b).

Early extraction of the ankylosed primary tooth and subsequent space management, particularly when patients are in the stages of early mixed dentition, is recommended (Savoldi et al. 2021). One of the principal causes of ankylosis in primary teeth is an asymmetric pattern of root resorption (Eşian et al. 2022).

To avoid dental retention, impacted teeth, and ankylosis of the primary dentition caused by the asymmetrical root resorption pattern of the second primary molar, a clinical-radiographic follow-up of the uneven resorption root is needed, and surgical intervention is indicated where necessary (Prove et al. 1992).

The null hypothesis was rejected, and the alternative hypothesis was accepted; there were asymmetrical root resorption patterns of the mandibular molars, and these differences were not due to chance.

## Limitations

This cross-sectional study cannot establish the cause of the asymmetric patterns of root resorption of the mandibular primary molars. Despite this, the findings of this study could be very useful to paediatric dentists and orthodontists and could help to determine any alterations in the pattern of physiological root resorption and to establish the normal length of the mandibular primary molars according to age and sex. The location of the premolars between the primary roots is associated with root resorption (Peretz et al. 2013). Some authors have reported that the pressure exerted by the permanent tooth plays a role in initiating resorption of the primary root, which begins when the primary root is close to the permanent tooth (Harokopakis-Hajishengallis 2007). It would be very interesting in future studies to design a longitudinal study that uses the same methodology and incorporates the localisation of the germ of the lower permanent premolar.

To limit the potential bias/errors associated with measurement tools, images with distortion and/or an irregular root apex were excluded. Different methods have been proposed for studying root resorption in primary dentition, most of which are qualitative methods (stages). In our study, we propose a quantitative method that clinics can easily apply.

## Conclusions

Delays in root resorption were detected in boys, whereas advances in root resorption were detected in girls. The root resorption pattern of the first mandibular primary molar was symmetrical; however, the pattern of the second mandibular primary molar was asymmetric, with the mesial root being resorbed first. Clinical and radiographic monitoring of mandibular primary molars with unevenly resorbed roots is recommended to avoid the complications associated with overretained molars.

## Supplementary Information

Below is the link to the electronic supplementary material.

Supplementary file1 (DOCX 1258 kb)

## Abbreviations

74: Lower left first temporary molar
75: Lower left second temporary molar
RR: Root resorption
LMR: Length of the mesial root
LDR: Length of the distal root
Fig: Figure
