# Correction to: Root resorption pattern and root length of mandibular primary molars in children: a cross-sectional radiographic study

**DOI:** 10.1007/s40368-025-01109-3

**Published:** 2025-09-18

**Authors:** C. S. Garcete Delvalle, M. Bruna del Cojo, M. R. Mourelle Martínez, M. J. De Nova García

**Affiliations:** 1 Department of Dental Clinical Specialties, School of Dentistry, Faculty of Dentistry, Complutense University of Madrid, 28040 Madrid, Spain; 2 Department of Dentistry, School of Medicine, CEU San Pablo University, 28668 Madrid, Spain

**Correction to: European Archives of Paediatric Dentistry** 10.1007/s40368-025-01052-3

The article [Root resorption pattern and root length of mandibular primary molars in children: a cross-sectional radiographic study], written by [C. S. Garcete Delvalle^1,2^ ⋅ M. Bruna del Cojo^2^ ⋅ M. R. Mourelle Martínez^1^ ⋅ M. J. De Nova García], was originally published Online First without Open access. After publication, the author decided to opt for Open Choice and to make the article an Open Access publication. Therefore, the copyright of the article has been changed to ©[The Author(s)] [2025] and this article is licensed under a Creative Commons Attribution 4.0 International License, which permits use, sharing,adaptation, distribution and reproduction in any medium or format, as long as you giveappropriate credit to the original author(s) and the source, provide a link to the CreativeCommons licence, and indicate if changes were made. The images or other third partymaterial in this article are included in the article’s Creative Commons licence, unlessindicated otherwise in a credit line to the material. If material is not included in the article’s Creative Commons licence and your intended use is not permitted by statutory regulation or exceeds the permitted use, you will need to obtain permission directly from the copyright holder. To view a copy of this licence, visit http://creativecommons.org/licenses/by/4.0/.

The original article has been corrected.

**Funding** Open Access provided thanks to the CRUE-CSIC agreement with Springer Nature.

