# Supplementary material for: Root resorption pattern and root length of mandibular primary molars in children: a cross-sectional radiographic study
Source: Eur Arch Paediatr Dent. 2025 May 7;26(5):977–86. doi: 10.1007/s40368-025-01052-3 (PMC12532672; doi:10.1007/s40368-025-01052-3)
Supplement: Supplementary file 1 — Supplementary file1 (DOCX 1258 kb) [file 40368_2025_1052_MOESM1_ESM.docx]

**Radiograph Calibration**

All radiographs were calibrated using the Pixel-stick program. The radiographic calibration sequence is explained below (Figures 1-4).

**Radiograph calibration:**

a. Define 124 pixels to equal 20 mm, 20 mm/124 pixels = 0.1613 mm per pixel.

b. Set the unit to mm.

c. Name the sample case number "control 1".

d. The zoom scale is 0.1613 mm, as obtained in step 1.


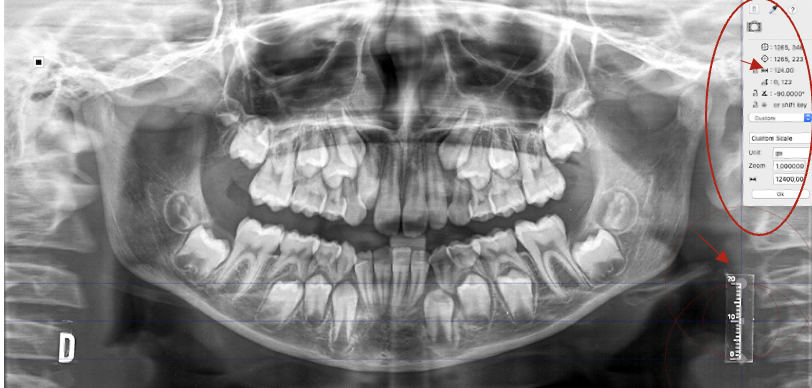


**Figure 1.** Radiographic calibration; first step: 124 pixels equal 20 mm, as shown in the image.

***
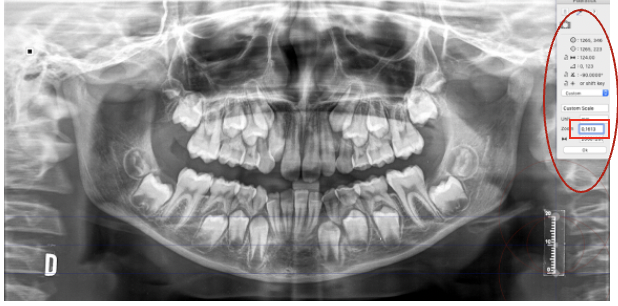
***

**Figure 2.** Radiographic calibration; second step: 124 pixels equal 20 mm; 20 mm/124 pixels indicates that each pixel represents 0.1613 mm. The unit is set to mm, as shown in the image.


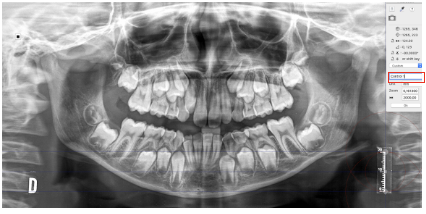


**Figure 3.** Radiographic calibration; third step: Name the case number. Set the unit to mm, as shown in the image.


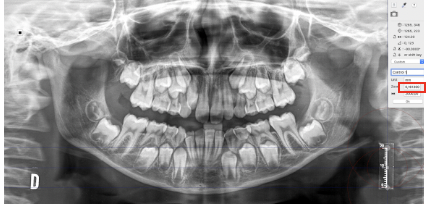


**Figure 4.** Radiographic calibration; step four: The zoom scale is 0.1613 mm, as obtained in step 1, as shown in the image.
